# COGNIZER: A Framework for Functional Annotation of Metagenomic Datasets

**DOI:** 10.1371/journal.pone.0142102

**Published:** 2015-11-11

**Authors:** Tungadri Bose, Mohammed Monzoorul Haque, CVSK Reddy, Sharmila S. Mande

**Affiliations:** Bio-Sciences R&D Division, TCS Innovation Labs, Tata Consultancy Services Limited, 54-B, Hadapsar Industrial Estate, Pune, 411013, Maharashtra, India; CSIR-Institute of Microbial Technology, INDIA

## Abstract

**Background:**

Recent advances in sequencing technologies have resulted in an unprecedented increase in the number of metagenomes that are being sequenced world-wide. Given their volume, functional annotation of metagenomic sequence datasets requires specialized computational tools/techniques. In spite of having high accuracy, existing stand-alone functional annotation tools necessitate end-users to perform compute-intensive homology searches of metagenomic datasets against "multiple" databases prior to functional analysis. Although, web-based functional annotation servers address to some extent the problem of availability of compute resources, uploading and analyzing huge volumes of sequence data on a shared public web-service has its own set of limitations. In this study, we present COGNIZER, a comprehensive stand-alone annotation framework which enables end-users to functionally annotate sequences constituting metagenomic datasets. The COGNIZER framework provides multiple workflow options. A subset of these options employs a novel directed-search strategy which helps in reducing the overall compute requirements for end-users. The COGNIZER framework includes a cross-mapping database that enables end-users to simultaneously derive/infer KEGG, Pfam, GO, and SEED subsystem information from the COG annotations.

**Results:**

Validation experiments performed with real-world metagenomes and metatranscriptomes, generated using diverse sequencing technologies, indicate that the novel directed-search strategy employed in COGNIZER helps in reducing the compute requirements without significant loss in annotation accuracy. A comparison of COGNIZER's results with pre-computed benchmark values indicate the reliability of the cross-mapping database employed in COGNIZER.

**Conclusion:**

The COGNIZER framework is capable of comprehensively annotating any metagenomic or metatranscriptomic dataset from varied sequencing platforms in functional terms. Multiple search options in COGNIZER provide end-users the flexibility of choosing a homology search protocol based on available compute resources. The cross-mapping database in COGNIZER is of high utility since it enables end-users to directly infer/derive KEGG, Pfam, GO, and SEED subsystem annotations from COG categorizations. Furthermore, availability of COGNIZER as a stand-alone scalable implementation is expected to make it a valuable annotation tool in the field of metagenomic research.

**Availability and Implementation:**

A Linux implementation of COGNIZER is freely available for download from the following links: http://metagenomics.atc.tcs.com/cognizer, https://metagenomics.atc.tcs.com/function/cognizer.

## Introduction

Recent advances in Next Generation Sequencing (NGS) techniques have facilitated large scale sequencing of genomes of microbial communities (also referred to as metagenomes) residing in diverse ecological niches. Sequencing data generated from metagenomes typically consists of millions of short nucleotide fragments (also referred to as 'reads'). One of the important steps in metagenomic data analysis pertains to estimation of the functional potential of various protein coding genes in the given dataset. Although homology-based approaches (like BLAST) provide the most accurate results with respect to functional annotation [1], the compute requirements of these approaches are enormous. Approaches employing additional heuristic steps (as compared to BLAST), have therefore been developed in order to address the problem of huge computational time required for protein database searches. For example, tools like RAPSearch [2] and PAUDA [3] employ 'inexact’ search techniques for significantly reducing computational costs that are typically associated with performing sequence similarity based searches. Although, these tools outperform BLAST-like approaches in terms of execution speed, the overall quality of results obtained with such tools, in several instances, is not at par with those obtained using non-heuristic approaches [3].

MEGAN [4] and FANTOM [5] are among the few applications that are available for stand-alone functional analysis of metagenomic reads. However, these GUI-based tools are primarily designed for analyzing pre-computed BLASTx results. End-users are required to perform compute intensive BLASTx searches prior to the use of these tools. For users having access to limited computing resources, this becomes a time consuming step. For example, a previous study had estimated that a computing facility with 1000-CPU compute-cluster would require approximately 30 days to complete a BLASTx search for a 20 GB metagenome against the NCBI-nr database [6].

Web-servers like MG-RAST [7], METAREP [8], CAMERA [9], CoMet [10], IMG/M [11], etc. provide an alternative means for end-users intending to perform functional annotation of metagenomic datasets. Although these web-servers provide a range of utilities for functional annotation, there is a limitation on the volume of reads that can be uploaded/analyzed by a specific end-user. Moreover, due to enormous demand, jobs submitted to these servers typically are processed based on priority-listing (which, in turn, is determined/governed by a variety of factors). In spite of appearing trivial, the stated limitations become a major hindrance for end-user's intending to analyze huge datasets. For instance, the total size of (Whole Genome Sequencing) datasets in recent metagenomic studies pertaining to diabetes [12,13] is in the range of 300–400 gigabytes. Uploading (and processing) such a huge volume of data to any of the public annotation servers may often prove to be infeasible for most end-users.

On a different note, protocols for functional annotation of individual sequences constituting metagenomic datasets are aimed at finding (a) COGs, i.e. the clusters of orthologous genes [14], (b) KEGG pathway mappings [15,16], (c) SEED subsystems [17], Gene Ontology (GO) [18], and (d) Pfam domain families [19]. Although existing stand-alone/web-based tools (mentioned above) provide functional annotations in terms of one or more of the above mentioned functional categories (viz., COG, KEGG, SEED, GO, Pfam), with the exception of the MG-RAST web-server, none of them provide functional annotations with respect to 'all' the mentioned categories. End-users of MG-RAST can obtain functional annotation of their uploaded datasets in terms of multiple functional databases like IMG, TrEMBL, PATRIC, SwissProt, Genbank NR, M5NR, SEED, RefSeq, eggNOG, KEGG, etc. However, as mentioned previously, the typical problems associated with uploading and analyzing huge volumes of sequenced data on a shared public web-service is definitely a major point of concern. In summary, the two major limitations of existing tools which are available for functional annotation of metagenomic datasets are (1) requirement of computationally expensive homology-based searches prior to use of stand-alone tools and (2) issues pertaining of usability (with respect to upload limit, analysis turn-around time, data privacy, etc.) of web-based services.

**Fig 1:**
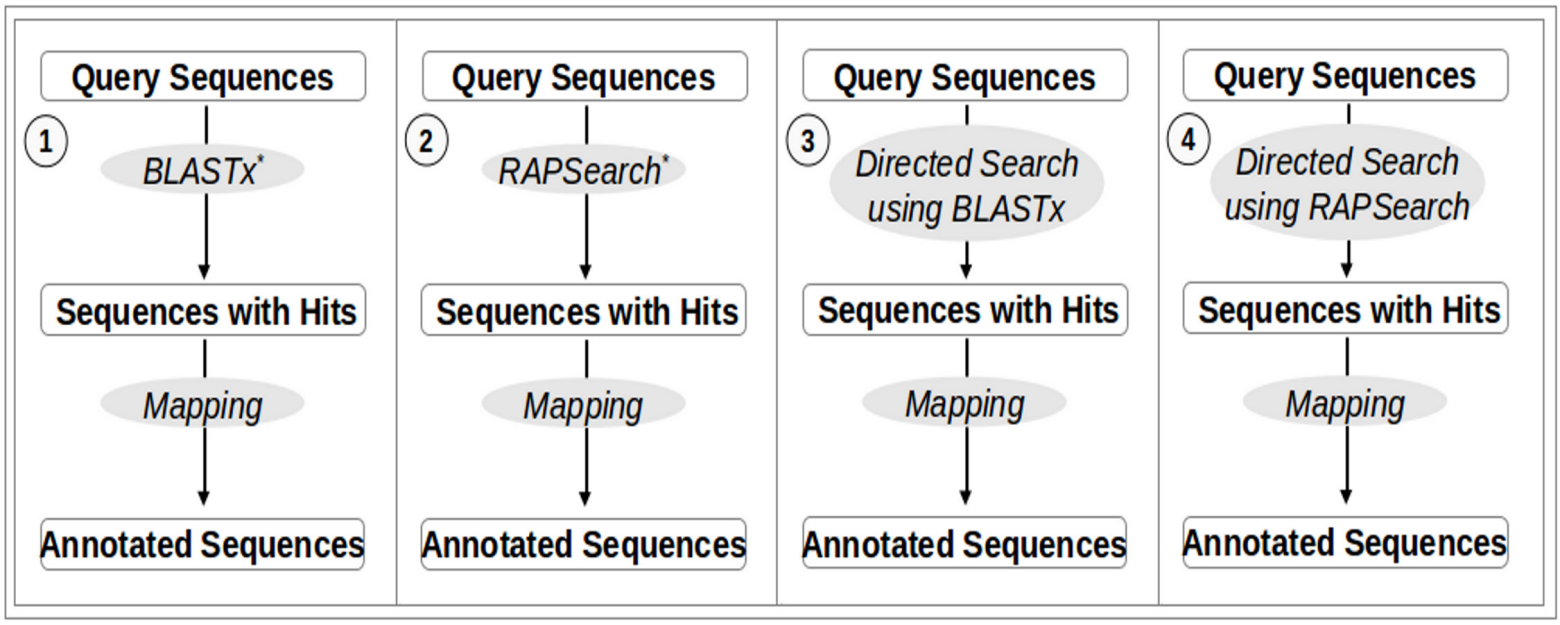
Workflow options in the COGNIZER workflow. A schematic representation of the four workflow options in the COGNIZER framework.

In this study, we present COGNIZER, a stand-alone framework that can be employed for functional annotation of metagenomic datasets. The framework provides four annotation workflow options (schematically represented in Fig 1). Each option employs a distinct 'homology-search' strategy requiring varying levels of compute power. These search options enable end-users to choose a homology-search protocol based on compute resources available at their disposal. End-users having access to huge compute power can employ the first and the second options i.e. the BLASTx and RAPSearch search options respectively. When there is limited availability of computing resources, users can deploy the COGNIZER framework with options 3 or 4. These two options employ a 'customized' COG database and use a novel directed-search strategy that can help in reducing the time required for database searches. The results of the ‘homology-search’ step (obtained using one of the above four options) are subsequently processed using the information present in COGNIZER’s customized cross-mapping database. This step enables end-users to obtain functional profiles of a given metagenome (with respect to multiple functional categories viz., COG, KEGG, SEED, GO, and Pfam) by performing a single database search.

## Methods

Fig 1 schematically represents the four annotation workflow options in the COGNIZER framework. Each option involves two phases, namely, a ‘homology-search’ phase and a 'mapping' phase, the latter phase being common to all four workflow options. In the 'homology-search' phase, sequences in the metagenomic dataset (to be analyzed) are queried against the COG database [14]. Options 1–4 differ with respect to the employed 'homology-search' strategy as well as the format of the COG database. The subsequent ‘mapping’ phase involves inferring KEGG, SEED, GO, and Pfam annotations from the COG annotations obtained in the ‘homology-search’ phase. A customized cross-mapping database is employed for this purpose. The following sections describe (a) the structure of the COG database utilized in each workflow option (b) the protocol used for creating the cross-mapping database, and (c) the overall algorithm employed (in each workflow) for obtaining functional annotations.

### (Customized) COG database

**Fig 2:**
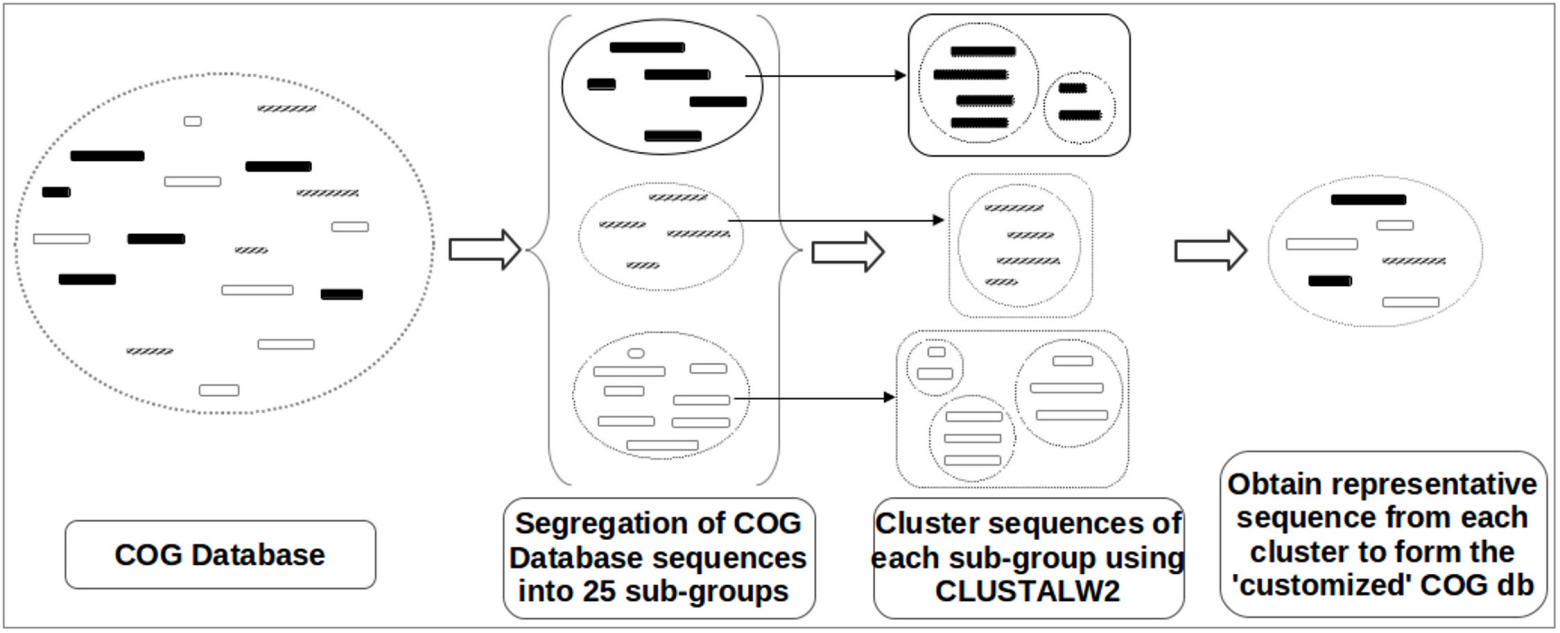
Creation of the 'customized' COG database. A schematic diagram illustrating the steps involved in the creation of the 'customized' COG database.

The COG database (available for download at http://www.ncbi.nlm.nih.gov/COG/) consists of approximately 200,000 protein sequences categorized into various COG groups. All protein sequences in the COG database are tagged to at least one of the 25 major functional COG categories [14]. This database, in its original form, is employed in options 1 and 2. For options 3 and 4, a 'customized' version of this database was created using the following procedure (Fig 2). Sequences in each functional COG category were first clustered using ClustalW2 [20] in default mode. This resulted in generating one or more clusters for each COG category. Subsequently, the longest protein sequence from each cluster was chosen and tagged to indicate the COG category to which it belonged. All (tagged) representative sequences were pooled together to form the 'customized' COG database. It may be noted that the customized database, thus generated, is approximately one-sixth in size as compared to the original COG database.

### Cross-mapping database

**Fig 3:**
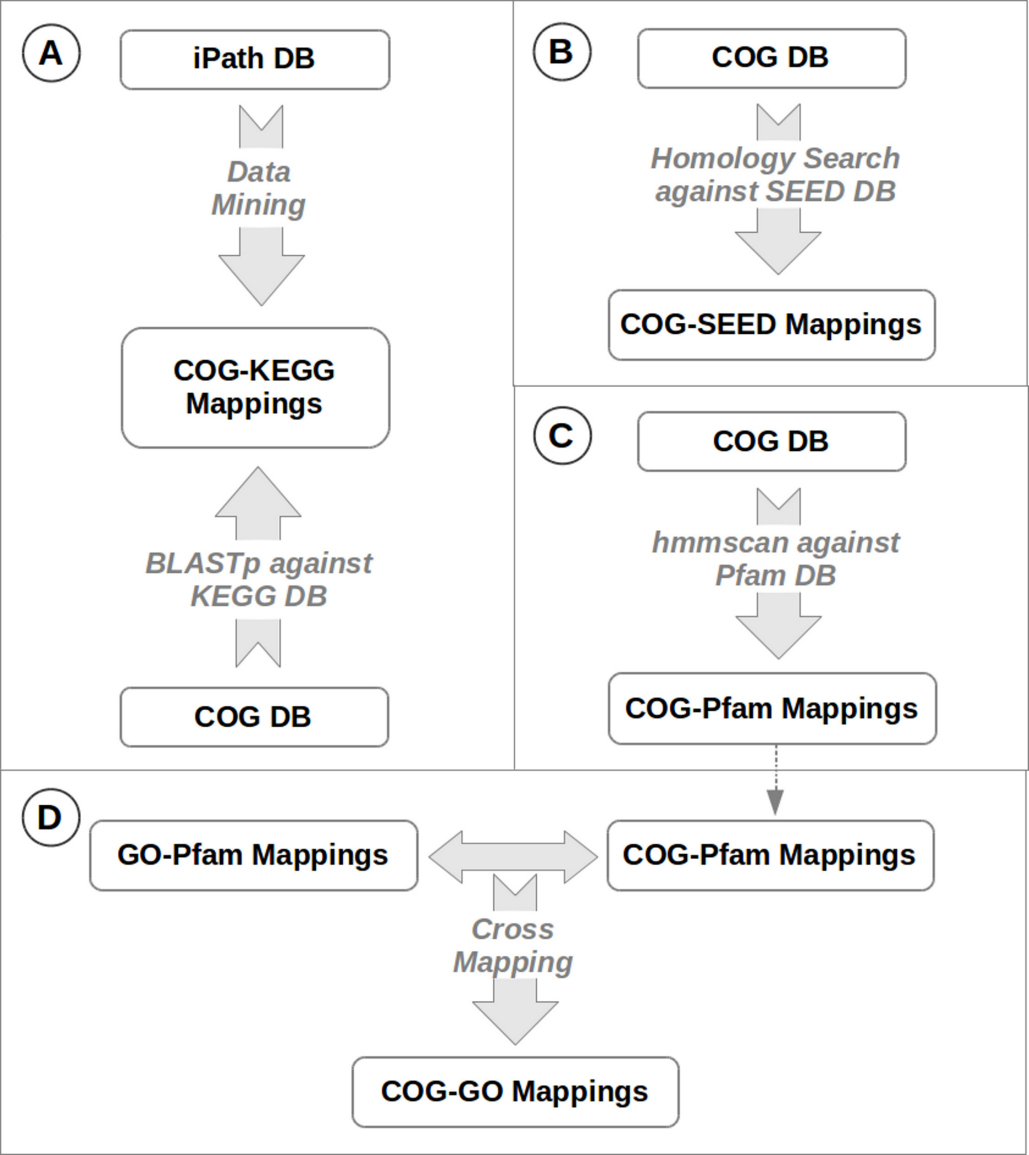
Procedure adopted for obtaining cross-mapping information. Procedure adopted for obtaining cross-mapping information amongst sequences in the COG and the other protein functional databases (KEGG, Pfam, GO and SEED).

A schematic representation of the steps involved in the creation of cross-mapping database is provided in Fig 3. The following sequence-search/data-mining approaches were employed for building a database containing cross-relationships between COG and other functional databases. Mapping between COG and KEGG identifiers were obtained by (a) mining COG-KEGG mapping information from the iPath database [21], and (b) performing BLAST-based searches of protein sequences from the COG database against the sequences from KEGG databases (using the CAMERA web-service). Information from both these sources was collated into a unified mapping file. In cases where the mapping between the two sources did not match, the mapping obtained using the BLAST approach was given preference. COG and Pfam identifier mappings were obtained by comparing COG database sequences against Pfam database. This comparison was done using hmmscan tool from the HMMER package [22]. Mappings between GO and PFAM annotations were obtained from the GO website (http://www.geneontology.org/external2go/pfam2go). These mappings were further processed to infer cross-relationships between GO and COG entries. Sequence homology searches were used for obtaining the SEED-COG mappings.

### Algorithm

Details of the four workflow options in COGNIZER method are as follows. In option 1, the BLASTx method is employed (in the homology-search phase) for querying reads constituting metagenomic datasets. The search is performed against all sequences in the COG database. The query sequence is assigned to the COG category that corresponds to the highest scoring BLASTx hit whose e-value is lower than a user-specified threshold. In the subsequent 'mapping' phase, for each query, functional annotation with respect to other databases viz., KEGG, SEED, GO, and Pfam is inferred using COGNIZER's cross-mapping database. Steps in option 2 are similar to those in option 1 except for the usage of RAPSearch algorithm instead of BLASTx (in the homology-search phase).

**Fig 4:**
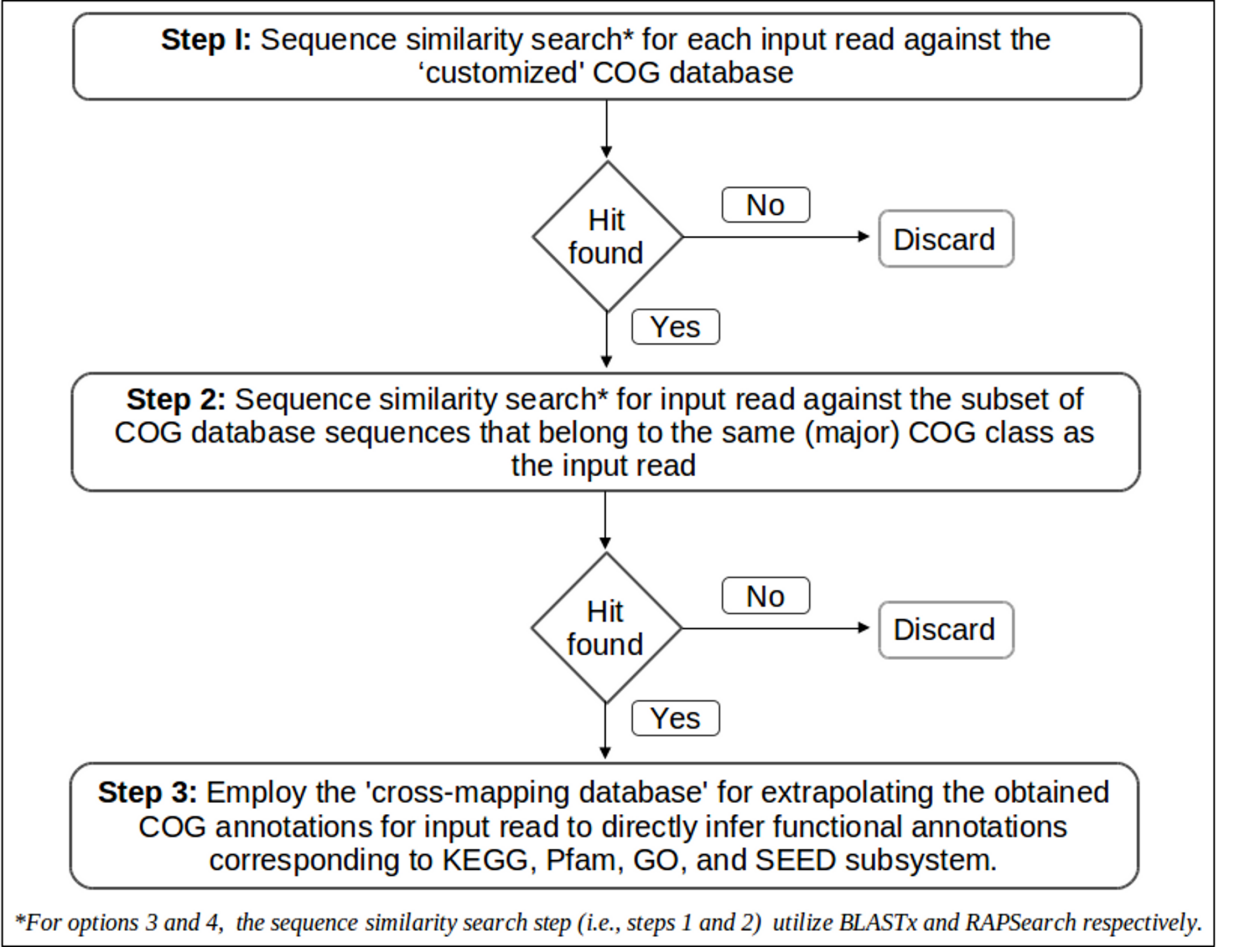
Workflows adopted in options 3 and 4 of the COGNIZER framework. A flow-diagram depicting the steps adopted in workflow options 3 and 4 of the COGNIZER framework.

Fig 4 illustrates the overall workflow for options 3 and 4 of COGNIZER. These options work in the following manner. In the first step, query sequences in the input metagenomic dataset are partitioned into subsets by performing similarity searches against sequences in the 'customized' COG database. This results in generating 25 query subsets, each subset consisting of sequences that have similarity to one of the 25 major COG categories. In other words, step 1 result in assigning a tentative high-level COG classification to each query sequence. In step 2, sequences in each query subset (tagged in step 1 to a COG category) are searched only against the subset of COG database sequences that belong to the same COG category. This directed-search approach (wherein subsets of query sequences are searched only against respective database partitions) therefore significantly reduces the search-space, and consequently decreases the overall compute requirements. At the end of step 2, sequences in the query dataset are annotated in terms of COG functional categories. In step 3, the pre-computed cross-mapping database is employed for extrapolating the obtained COG annotations to directly infer functional annotations corresponding to KEGG, Pfam, GO, and SEED subsystem databases. This extrapolation step does not involve compute intensive (alignment-based) searches. It may be noted that while option 3 employs BLASTx, option 4 uses the RAPSearch algorithm in steps 1 and 2.

### Validation datasets

The performance of the COGNIZER framework was evaluated using 21 real-world datasets comprising (a) 3 hyper-saline saltern metagenomes [23] (b) 7 metatranscriptomic datasets [24] (c) 2 gut metagenomes from healthy and malnourished children [25] (d) 8 oral metagenomes [26], and (e) acid mine drainage (AMD) metagenome [27]. These datasets were chosen since they fairly represented the typical characteristics of sequence data obtained using three well-known sequencing technologies viz. Illumina, Roche-454 and Sanger.

### Validation strategy

COGNIZER employs a cross-mapping database for deriving functional annotations corresponding to KEGG, SEED, GO, and Pfam from the COG annotations. It is therefore essential to verify the accuracy of the derived annotations. Furthermore, since some options available in COGNIZER utilize a ‘customized’ COG database (for reducing execution time), validation of the COG annotations obtained using these options is also required. Therefore, COG, KEGG, SEED, and Pfam annotations (for sequences in individual datasets) were obtained by ‘directly’ performing requisite homology searches against all sequences in the respective functional database. Annotations obtained in this manner were considered as ‘benchmarks’. Given that GO mappings in the COGNIZER framework were directly obtained from the GO website, the benchmark validation procedure for GO annotations was not performed. The results obtained with the four workflow options (in COGNIZER) were then compared against the pre-computed benchmark values.

The performance of the COGNIZER framework was evaluated in terms of (a) execution time (b) Positive Predictive Value (PPV), and (c) Negative Predictive Value (NPV). The latter two metrics were calculated as follows:

PPV=number of true positives/(number of true positives+number of false positives)

NPV=number of true negatives/(number of true negatives+number of false negatives)

## Results and Discussion

The performance of various options of COGNIZER, in terms of PPV and NPV, is summarized in Table 1. Results obtained with option 1 of COGNIZER (i.e. BLASTx followed by mapping step) indicate high (average >0.98) PPV and NPV values as compared to the benchmark values. These results confirm the reliability of the cross-mapping database employed in the COGNIZER framework. A relatively lower accuracy (average >0.94) is observed with option 2 which adopts the RAPSearch algorithm in the search phase. This is expected since RAPSearch employs a heuristic 'reduced amino acid alphabet' based search approach for reducing the associated computational costs. In this context, it is interesting to note that the marginal gain in annotation accuracy of BLASTx (option 1) over RAPSearch (option 2) comes at a huge computational cost. As observed in Table 2, RAPSearch takes only one-fourth of the processing time required by BLASTx.

**Table 1:** Evaluation of COGNIZER's annotation results in terms of positive predictive value (PPV) and negative predictive value (NPV).

Sequencing Platform	Dataset^#^	Option	COG Annotation		KEGG Annotation		Pfam Annotation		SEED annotation	
			PPV	NPV	PPV	NPV	PPV	NPV	PPV	NPV
Illumina (Average Read Length: 100 bp)	High Salt Metagenome (35446)	1	1.00	1.00	0.99	0.99	0.99	0.99	0.99	0.99
		2	0.97	1.00	0.96	0.99	0.96	0.99	0.96	0.99
		3	0.77	0.98	0.75	0.97	0.75	0.97	0.79	0.98
		4	0.76	0.98	0.74	0.97	0.75	0.96	0.78	0.97
	Medium Salt Metagenome (38929)	1	1.00	1.00	0.99	0.99	0.99	0.99	0.99	0.99
		2	0.98	1.00	0.97	0.99	0.97	0.99	0.96	0.99
		3	0.79	0.97	0.76	0.96	0.78	0.96	0.80	0.97
		4	0.78	0.96	0.76	0.95	0.78	0.95	0.80	0.96
	Low Salt Metagenome (34296)	1	1.00	1.00	0.99	0.99	0.99	0.99	0.99	0.99
		2	0.98	1.00	0.97	0.99	0.97	0.99	0.96	0.99
		3	0.81	0.99	0.78	0.98	0.79	0.98	0.79	0.98
		4	0.79	0.99	0.77	0.97	0.76	0.98	0.77	0.98
Illumina Metatranscriptomic Datasets (Average Read Length: 209 bp)	SRR397002 (587272)	1	1.00	1.00	0.99	0.99	0.99	0.99	0.99	0.99
		2	0.95	0.97	0.90	0.89	0.94	0.95	0.93	0.93
		3	0.92	0.94	0.82	0.85	0.90	0.92	0.86	0.88
		4	0.92	0.93	0.79	0.79	0.86	0.91	0.83	0.84
	SRR397004 (570339)	1	1.00	1.00	0.99	0.99	0.99	0.99	0.99	0.99
		2	0.96	0.96	0.94	0.92	0.93	0.94	0.92	0.94
		3	0.93	0.93	0.86	0.90	0.87	0.90	0.82	0.90
		4	0.90	0.91	0.83	0.84	0.85	0.83	0.76	0.86
	SRR397074 (564583)	1	1.00	1.00	0.99	0.99	0.99	0.99	0.99	0.99
		2	0.96	0.98	0.91	0.88	0.89	0.96	0.94	0.94
		3	0.92	0.96	0.89	0.86	0.79	0.92	0.82	0.90
		4	0.90	0.92	0.83	0.81	0.75	0.84	0.79	0.86
	SRR397076 (561040)	1	1.00	1.00	0.99	0.99	0.99	0.99	0.99	0.99
		2	0.95	0.97	0.92	0.90	0.93	0.92	0.92	0.90
		3	0.93	0.95	0.87	0.91	0.89	0.90	0.88	0.84
		4	0.91	0.91	0.80	0.86	0.85	0.88	0.83	0.79
	SRR397079 (596020)	1	1.00	1.00	0.99	0.99	0.99	0.99	0.99	0.99
		2	0.96	0.97	0.92	0.89	0.94	0.96	0.96	0.93
		3	0.94	0.94	0.86	0.88	0.91	0.92	0.90	0.87
		4	0.92	0.90	0.81	0.82	0.87	0.89	0.78	0.82
	SRR397146 (583386)	1	1.00	1.00	0.99	0.99	0.99	0.99	0.99	0.99
		2	0.97	0.97	0.92	0.91	0.93	0.92	0.93	0.94
		3	0.94	0.94	0.89	0.90	0.88	0.89	0.84	0.90
		4	0.90	0.91	0.81	0.87	0.87	0.82	0.81	0.87
	SRR397148 (564583)	1	1.00	1.00	0.99	0.99	0.99	0.99	0.99	0.99
		2	0.96	0.97	0.92	0.90	0.95	0.84	0.90	0.88
		3	0.92	0.94	0.90	0.85	0.86	0.80	0.84	0.87
		4	0.91	0.91	0.84	0.82	0.82	0.76	0.81	0.80
Roche 454 (Average Read Length: 350 bp)	Malnourished Child Gut Metagenome (1501481)	1	1.00	1.00	0.99	0.99	0.99	0.99	0.99	0.99
		2	0.87	0.99	0.81	0.95	0.85	0.98	0.80	0.96
		3	0.88	0.96	0.85	0.81	0.85	0.92	0.81	0.87
		4	0.67	0.98	0.60	0.88	0.64	0.95	0.55	0.91
	Healthy Child Gut Metagenome (1501481)	1	1.00	1.00	0.75	0.94	0.77	0.94	0.79	0.95
		2	0.86	0.99	0.96	0.98	0.96	0.98	0.95	0.98
		3	0.89	0.97	0.85	0.98	0.86	0.98	0.87	0.98
		4	0.66	0.98	0.98	0.98	0.98	0.98	0.98	0.98
Roche 454 Oral Metagenomic samples (Average Read Length 400 bp)	4447101.3.6941 (295072)	1	1.00	1.00	0.99	0.99	0.99	0.99	0.99	0.99
		2	0.99	0.99	0.94	0.96	0.97	0.96	0.97	0.96
		3	0.97	0.98	0.91	0.92	0.94	0.94	0.79	0.94
		4	0.96	0.96	0.84	0.89	0.91	0.92	0.79	0.89
	4447102.3.6942 (244881)	1	1.00	1.00	0.99	0.99	0.99	0.99	0.99	0.99
		2	0.98	0.99	0.95	0.96	0.97	0.96	0.92	0.97
		3	0.96	0.97	0.83	0.92	0.95	0.94	0.78	0.94
		4	0.96	0.95	0.76	0.88	0.92	0.83	0.77	0.92
	4447103.3.6943 (464594)	1	1.00	1.00	0.99	0.99	0.99	0.99	0.99	0.99
		2	0.99	0.99	0.95	0.94	0.97	0.98	0.94	0.95
		3	0.96	0.97	0.91	0.89	0.93	0.96	0.88	0.90
		4	0.95	0.93	0.85	0.89	0.90	0.95	0.82	0.86
	4447192.3.7032 (204218)	1	1.00	1.00	0.99	0.99	0.99	0.99	0.99	0.99
		2	0.99	0.99	0.95	0.95	0.97	0.95	0.92	0.94
		3	0.97	0.96	0.89	0.88	0.95	0.92	0.83	0.92
		4	0.96	0.92	0.82	0.84	0.93	0.74	0.77	0.88
	4447903.3.7714 (306740)	1	1.00	1.00	0.99	0.99	0.99	0.99	0.99	0.99
		2	0.98	0.98	0.94	0.96	0.97	0.96	0.91	0.96
		3	0.96	0.94	0.90	0.91	0.93	0.93	0.87	0.94
		4	0.95	0.91	0.86	0.88	0.91	0.89	0.79	0.89
	4447943.3.7744 (339503)	1	1.00	1.00	0.99	0.99	0.99	0.99	0.99	0.99
		2	0.99	0.97	0.93	0.93	0.97	0.97	0.90	0.96
		3	0.97	0.95	0.89	0.89	0.95	0.92	0.82	0.94
		4	0.96	0.93	0.83	0.83	0.92	0.91	0.76	0.91
	4447970.3.1 (70503)	1	1.00	1.00	0.99	0.99	0.99	0.99	0.99	0.99
		2	0.99	0.98	0.93	0.94	0.97	0.97	0.89	0.94
		3	0.96	0.96	0.88	0.90	0.95	0.93	0.81	0.94
		4	0.96	0.91	0.84	0.86	0.92	0.92	0.70	0.89
	4447971.3.6 (97722)	1	1.00	1.00	0.99	0.99	0.99	0.99	0.99	0.99
		2	0.98	0.97	0.93	0.93	0.98	0.97	0.92	0.93
		3	0.97	0.95	0.91	0.89	0.92	0.94	0.85	0.93
		4	0.96	0.92	0.88	0.84	0.89	0.93	0.78	0.88
Sanger (Average Read Length: 1000bp)	Acid Mine Drainage Metagenome (180713)	1	1.00	1.00	0.99	0.99	0.99	0.99	0.99	0.99
		2	0.98	0.78	0.93	0.74	0.96	0.72	0.90	0.87
		3	0.95	0.82	0.65	0.79	0.82	0.73	0.63	0.90
		4	0.95	0.68	0.72	0.66	0.86	0.63	0.69	0.80

# Number within brackets indicates the total number of reads in each of the validation datasets. For oral metagenomes, MG-RAST ids are provided as dataset identifiers.

**Table 2:** Comparison of computing time required by different options in the COGNIZER framework.

Sequencing Platform	Dataset^#^	Option	Time (secs)*	Percentage Reduction in time as compared to option 1
Illumina (Average Read Length: 100 bp)	High Salt Metagenome (35446)	1	507	-
		2	120	76.33
		3	378	25.44
		4	111	78.11
	Medium Salt Metagenome (38929)	1	551	-
		2	125	77.31
		3	392	28.86
		4	111	79.85
	Low Salt Metagenome (34296)	1	398	-
		2	94	76.38
		3	287	27.89
		4	90	77.39
Illumina Meta-transcriptomic Datasets (Average Read Length: 209 bp)	SRR397002 (587272)	1	15272	-
		2	2772	81.85
		3	9274	39.27
		4	1136	92.56
	SRR397004 (570339)	1	15491	-
		2	2814	81.83
		3	8949	42.23
		4	1157	92.53
	SRR397074 (564583)	1	17627	-
		2	3579	79.70
		3	9536	45.90
		4	1595	90.95
	SRR397076 (561040)	1	17979	-
		2	3535	80.34
		3	9282	48.37
		4	1583	91.20
	SRR397079 (596020)	1	14411	-
		2	3039	78.91
		3	7294	49.39
		4	1759	87.79
	SRR397146 (583386)	1	14664	-
		2	3375	76.98
		3	8261	43.66
		4	1732	88.19
	SRR397148 (564583)	1	13752	-
		2	2986	78.29
		3	7950	42.19
		4	1697	87.66
Roche 454 (Average Read Length: 350 bp)	Malnourished Child Gut Metagenome (1501481)	1	56700	-
		2	17040	69.95
		3	40620	28.36
		4	9840	82.65
	Healthy Child Gut Metagenome (1501481)	1	48540	-
		2	13800	71.57
		3	36900	23.98
		4	8220	83.07
Roche 454 Oral Metagenomic samples (Average Read Length 400 bp)	4447101.3.6941 (295072)	1	19085	-
		2	6048	68.31
		3	12525	34.37
		4	3706	80.58
	4447102.3.6942 (244881)	1	14780	-
		2	4466	69.78
		3	9224	37.59
		4	2728	81.54
	4447103.3.6943 (464594)	1	30130	-
		2	10084	66.53
		3	20716	31.24
		4	6048	79.93
	4447192.3.7032 (204218)	1	11772	-
		2	3621	69.24
		3	8027	31.81
		4	2382	79.77
	4447903.3.7714 (306740)	1	18253	-
		2	5713	68.70
		3	12108	33.67
		4	3725	79.59
	4447943.3.7744 (339503)	1	21127	-
		2	6852	67.57
		3	13941	34.01
		4	4143	80.39
	4447970.3.1 (70503)	1	4087	-
		2	1307	68.02
		3	2763	32.40
		4	882	78.42
	4447971.3.6 (97722)	1	5652	-
		2	1893	66.51
		3	3803	32.71
		4	1168	79.33
Sanger (Average Read Length: 1000bp)	Acid Mine Drainage Metagenome (180713)	1	21680	-
		2	7100	67.25
		3	9020	58.39
		4	5650	73.94

* All validation experiments were performed on a CentOS (ver. 6.3) server having 64 Intel Xeon dual-core 2.3 Ghz processors and 128 GBs of RAM. Individual options of COGNIZER were executed using 32 CPU threads.

^#^ Number within brackets indicates the total number of reads in each of the validation datasets. For oral metagenomes, MG-RAST ids are provided as dataset identifiers.

In spite of employing a directed search strategy against a customized (reduced) COG database, the PPV and NPV values obtained with options 3 and 4 of COGNIZER (in majority of cases) are observed to be in the range of 0.76–0.95 (Table 1). Significantly, for most datasets having sequences with read-length greater than 300 bp, the mean PPV and NPV values of options 3 and 4 are observed to relatively higher than those obtained with datasets with shorter reads. The probable reason behind this observation is as follows. Sequence fragments of longer lengths are more likely to generate relatively robust alignments thereby decreasing the likelihood of predicting a false positive outcome. Furthermore, proteins typically comprise of multiple functional domains. Consequently, the probability of encompassing information corresponding to multiple protein domains is relatively higher for longer sequence fragments. The slight improvement in results obtained with datasets having higher mean sequence lengths (typically those from the 454-Roche and the Sanger sequencing technology) are a reflection of the same. Given that most of the currently available sequencing technologies have the capability to generate reads with length of at least 250 bp, the results obtained with options 3 and 4 (with datasets having sequences of length 300 and above) assume relevance in the present context. With respect to processing time, options 3 and 4 are observed to outperform options 1 and 2 respectively (Table 2), thereby reflecting the utility of the directed search strategy in reducing the computational costs.

**Fig 5:**
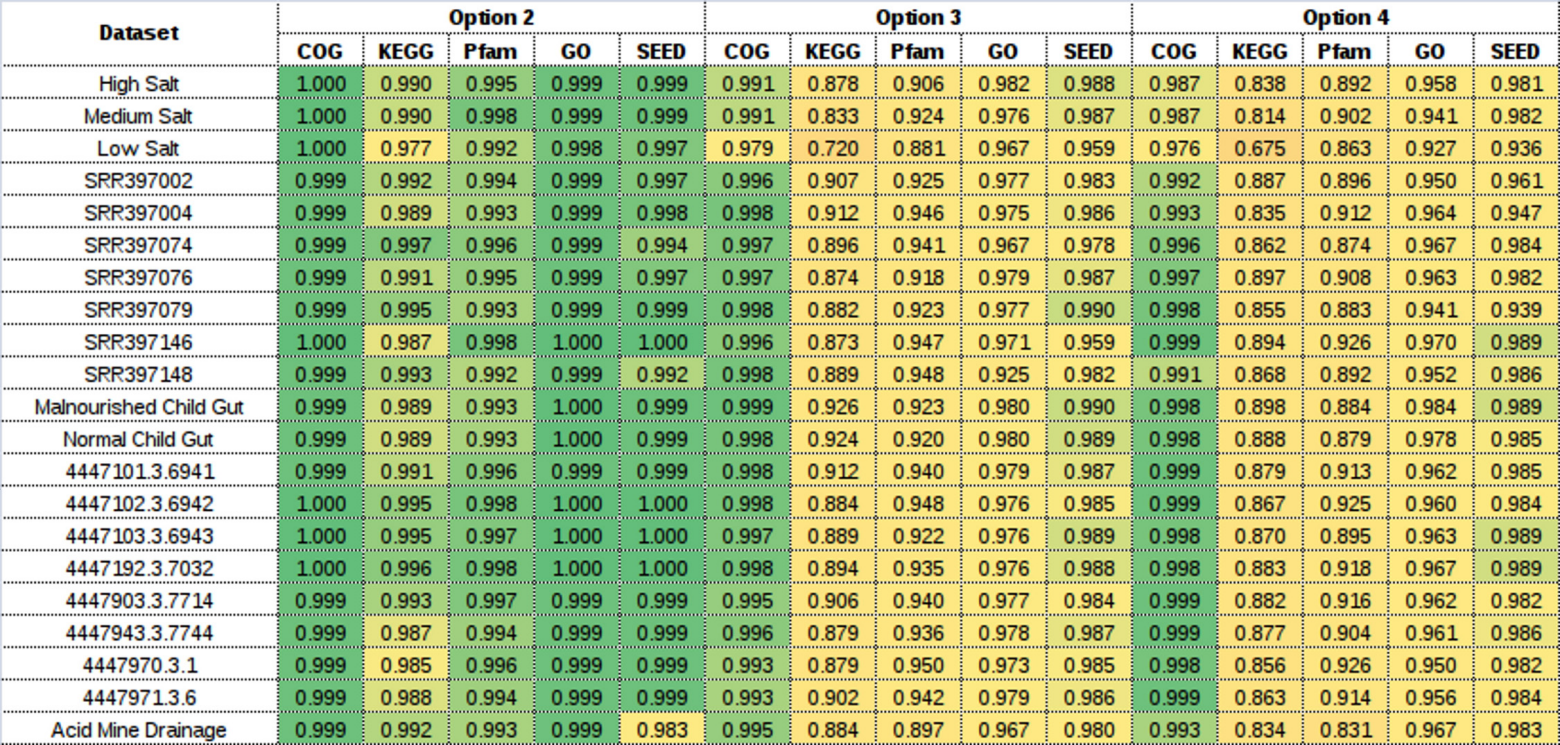
Correlation between prediction results obtained using option 1 and those obtained using the other three options in the COGNIZER framework. A heat map of the correlation coefficients between the annotations obtained using option 1 and the other three options of COGNIZER framework. Pearson correlation coefficients were obtained with a p-value confidence of <0.00001. In option 1, the BLASTx method is employed (in the homology-search phase) for querying reads constituting metagenomic datasets. The search is performed against all sequences in the COG database. In the subsequent 'mapping' phase, for each query, functional annotations are inferred using COGNIZER's cross-mapping database. In option 2 the RAPSearch algorithm is used instead of BLASTx (in the homology-search phase). Option 3 and 4 are analogous to options 1 and 2 respectively, except that a reduced/customised COG database is used during the homology-search phase.

A heat map depicting correlation between the annotation results obtained using option 1 and those obtained using the other three options of COGNIZER is presented in Fig 5. In summary, validation results provided in Tables 1 and 2 along with results depicted in Fig 5 indicate that options 2 and 4 represent an optimal trade-off between execution time and annotation accuracy. It is pertinent to note here that all workflow options of COGNIZER (including options 2 and 4) rely on the cross-mapping database for deriving annotations pertaining to different functional databases (KEGG, SEED, GO, and Pfam) using a single homology search against the COG database. Consequently, this database constitutes a key component of this stand-alone functional annotation framework. Interestingly, results presented in Tables 1 and 2 and Fig 5 further indicate that the drop in annotation accuracy with options 2–4 (as compared to option 1) is more or less consistent across various datasets (irrespective of read length). This is expected given that options 2–4 involve additional heuristic features. Overall, the annotation accuracy appears to be dependent on both query sequence length as well as the heuristic option employed.

COGNIZER relies primarily on the COG database. The main reason for choosing the COG database is as follows. The COG database, comprising of approximately 200,000 protein sequences, is a relatively smaller database as compared to other protein databases. For instance, the Pfam database, a collection of HMM profiles (not actual protein sequences), exceeds 1.2 GB (as compared to 70 MB of the COG database). Furthermore, the COG database captures most of the known protein functional categories. A recent review has reported that, in spite of the difference in database sizes, the quality of annotation (i.e. categorization of protein sequences into functional classes) obtained using the COG and the RefSeq databases are comparable [1]. The directed-search approach employed in COGNIZER therefore helps in further reducing the computing requirements without substantial loss in annotation accuracy. It is pertinent to note here that in all validation experiments, the peak memory requirement of COGNIZER rarely exceeded 500 MBs of RAM usage.

Usage of options 3 and 4, employing a reduced COG database in conjunction with cross-mapping framework, is logically expected to result in some degree of loss in annotation accuracy. Not withstanding this fact, the availability of compute resources is expected to drive/dictate the choice of options by end-users. For instance, analysis of the diabetes datasets [12,13] (having a cumulative volume of 300–400 gigabytes) is expected to entail huge compute resources and time, and hence usage of option 4 appears to be the logical choice. In spite of some loss in annotation accuracy, the results generated using this option would help in obtaining macro-level profiles (corresponding to various functional aspects) of these metagenomes. Results presented in this study with varied datasets (with all four options) are expected to serve as a guideline for end-users to decide upon an acceptable trade-off between execution time and prediction accuracy based on the compute resources available at their end.

The architectures of existing protein databases (e.g. Pfam, COG, SEED, etc.) are not similar. While the COG database is based on the evolutionary relatedness of genes/proteins from different organisms, the Pfam database contains information pertaining to protein domains and families. The KEGG annotations, in contrast, are employed for estimating metabolic pathways that are functional among the organisms constituting a metagenome. With its cross-mapping database, COGNIZER enables obtaining multiple functional annotations using a single homology search.

A recently published study [3], has proposed an alternate approach (PAUDA) for annotating metagenomic datasets against protein databases. Although PAUDA outperforms all four options (available in the COGNIZER framework) in terms of operational speed, the authors report that the tool is able to achieve an assignment rate of only 33% as compared to BLASTx. The NPV of PAUDA is therefore expected to be very low. In contrast, results obtained with all four options of COGNIZER demonstrate significantly relative higher NPV values. In addition, the cross-mapping utility in the COGNIZER framework enables end-users to obtain multiple functional annotations (using a single homology search) in a time efficient manner. The COGNIZER framework therefore provides significant value addition to researchers working in the field of metagenomics and metatranscriptomics.

COGNIZER software has been implemented as a generic framework. In principle, any sequence alignment tool can be integrated within this framework for performing homology searches of query sequences against sequences in the COG database (or its customized variant). In the present implementation, sequence alignment tools which are compatible with both 32-bit and 64-bit system architectures were included. Given this, the present distribution of COGNIZER does not integrate DIAMOND [28]—a recently published homology search tool (with a 64-bit implementation) that can perform sequence alignments at a pace that drastically exceeds any of the tools currently implemented in the COGNIZER framework. In spite of its superior processing speed, experiments performed with the a subset of same datasets (used for evaluating the performance of COGNIZER) indicated a lower sensitivity/specificity of DIAMOND as compared to that obtained with RAPSearch and/or BLASTx (S1 Fig). However, as mentioned above, end-users intending to harness the rapid processing speed of DIAMOND can easily integrate this tool into the COGNIZER framework.

## Conclusion

Validation results demonstrate that the COGNIZER framework is capable of comprehensively annotating any metagenomic or metatranscriptomic dataset (from varied sequencing platforms) in functional terms. Multiple search options in COGNIZER provide the flexibility for choosing a homology search protocol based on available compute resources. The cross-mapping database in COGNIZER enables end-users to directly infer/derive Pfam, KEGG, GO, and SEED subsystem annotations from COG categorizations. This cross-mapping greatly increases the utility of COGNIZER. Furthermore, availability of COGNIZER as a stand-alone (scalable) implementation is expected to make it a valuable annotation tool in the field of metagenomic and metatranscriptomic research.

## Supporting Information

S1 Fig Comparison of the performance of RAPSearch and DIAMOND. Comparative analysis of the specificity and sensitivity of RAPSearch and DIAMOND in comparison to BLASTX. The analysis was performed at an e-value cut-off of 0.00001.
(TIF)
