# Supplementary figures and images for: COGNIZER: A Framework for Functional Annotation of Metagenomic Datasets
Source: PLoS One. 2015 Nov 11;10(11):e0142102. doi: 10.1371/journal.pone.0142102 (PMC4641738; doi:10.1371/journal.pone.0142102)

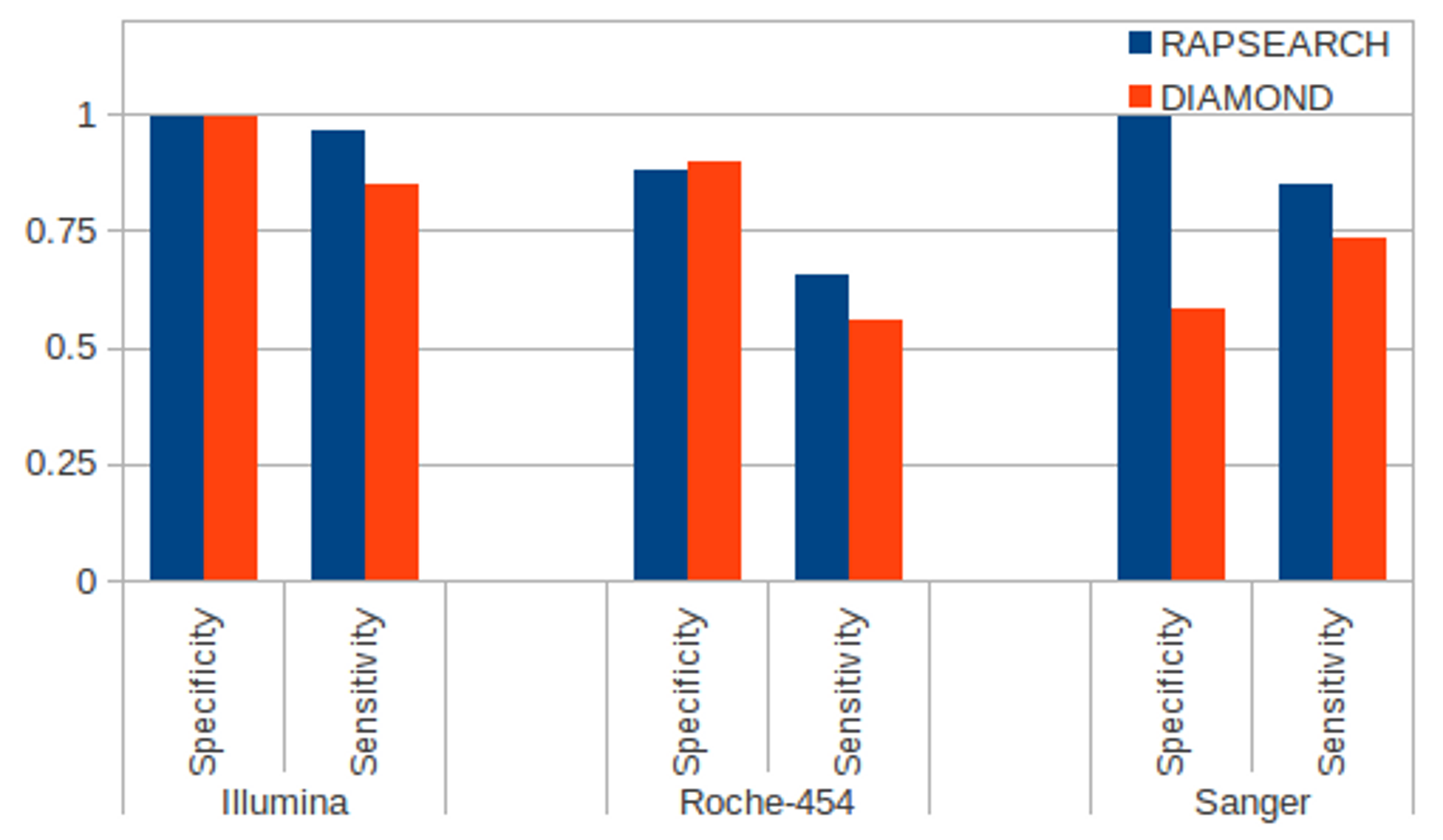

Supplement: S1 Fig — Comparative analysis of the specificity and sensitivity of RAPSearch and DIAMOND in comparison to BLASTX. The analysis was performed at an e-value cut-off of 0.00001. (TIF) [file pone.0142102.s001.tif]
